# Identification of key genes predicting the efficacy of mepolizumab in the treatment of severe eosinophilic asthma

**DOI:** 10.1038/s41598-025-23443-8

**Published:** 2025-11-12

**Authors:** Peng Xinmin, Cheng Jinxia, Li Fengyuan

**Affiliations:** Department of Respiratory and Critical Care Medicine, Nanchang First Hospital, Nanchang, China

**Keywords:** Severe eosinophilic asthma, Mepolizumab, Bioinformatics, Biomarkers, Computational biology and bioinformatics, Immunology

## Abstract

Severe eosinophilic asthma is a treatment-resistant subtype that remains poorly controlled with conventional therapies. According to the latest European Respiratory Society (ERS) guidelines, anti-interleukin-5 (anti-IL-5) biologics such as mepolizumab are recommended for these patients. However, not all individuals respond favorably to mepolizumab. This study aimed to identify gene expression biomarkers predictive of therapeutic response to mepolizumab, facilitating early identification of likely responders. Transcriptomic data (GSE274410) were obtained from the Gene Expression Omnibus (GEO) database. Differentially expressed genes (DEGs) between responder and non-responder groups were identified. Functional enrichment was conducted using Gene Ontology (GO) and Kyoto Encyclopedia of Genes and Genomes (KEGG) gene sets via gene set enrichment analysis (GSEA). Protein–protein interaction (PPI) networks were constructed using the STRING database, and hub genes were identified with the cytoHubba plugin in Cytoscape. Box plots were used to visualize gene expression patterns. FOS and CXCL3 were significantly downregulated in the responder group. Their predictive value was further assessed using receiver operating characteristic (ROC) curve analysis. These findings suggest that FOS and CXCL3 may serve as promising biomarkers for predicting response to mepolizumab and may aid in stratifying patients with severe eosinophilic asthma for individualized biologic therapy.

**Supplementary Information:**

The online version contains supplementary material available at 10.1038/s41598-025-23443-8.

## Introduction

Asthma is a heterogeneous respiratory disease characterized by chronic airway inflammation. It is commonly manifested by wheezing, shortness of breath, chest tightness, and coughing, with one or more symptoms often predominating^1^. Asthma can be phenotypically classified into eosinophilic, neutrophilic, mixed granulocytic, and paucigranulocytic subtypes based on differential cell counts in induced sputum^2^. Epidemiological data indicate a significant increase in the prevalence of asthma in China, rising from 0.69% in 1984 to 5.30% in 2021. Projections suggest that this figure may reach 9.76% by 2050^3^. The growing prevalence of asthma imposes a substantial burden on China’s healthcare and economic systems, with severe asthma accounting for a major proportion of this burden. Severe asthma is defined as asthma that remains uncontrolled despite treatment with high-dose inhaled corticosteroids in combination with long-acting β₂-agonists (ICS-LABA), or that requires ongoing therapy with high-dose ICS-LABA or biologic agents to prevent exacerbations^1^. According to the European Respiratory Society/American Thoracic Society (ERS/ATS) guidelines, anti–interleukin-5 (anti–IL-5) biologic agents are recommended for the treatment of severe eosinophilic asthma^4^.

Eosinophilic asthma is characterized by elevated eosinophil levels in induced sputum and peripheral blood. Evidence suggests that increased eosinophil counts in the peripheral blood or airways are associated with greater asthma severity^5^. Interleukin-5 (IL-5) is a cytokine that promotes eosinophil proliferation^6^, thereby contributing to the worsening of asthma symptoms. Mepolizumab is an anti–IL-5 monoclonal antibody that inhibits IL-5, thereby suppressing eosinophil proliferation and activation, ultimately reducing airway inflammation^7^. Current studies indicate that mepolizumab treatment for severe eosinophilic asthma can reduce acute exacerbations by nearly 50%, decrease hospitalization rates by approximately 30%, and improve lung function^8^. However, not all patients with eosinophilic asthma respond to mepolizumab treatment, underscoring the importance of identifying and selecting specific subpopulations that are most likely to benefit from this therapy.

In recent years, bioinformatics has gained increasing prominence and has yielded significant advances in disease prediction and diagnosis. This study aims to leverage bioinformatics data to identify early predictive biomarkers of mepolizumab efficacy in the treatment of severe eosinophilic asthma.

## Materials and methods

### Data collection and processing

The high-throughput sequencing dataset GSE274410 was downloaded from the Gene Expression Omnibus (GEO) database (https://www.ncbi.nlm.nih.gov/geo/). This dataset is based on the GPL24676 platform, and detailed descriptions of the dataset and its processing methods are provided in the study by Kamini Rakkar et al.^9^. The GSE274410 dataset comprises 54 nasal swab samples from 27 patients, including 27 samples collected before treatment and 27 samples collected three months after treatment. For this study, the pre-treatment nasal swab samples were selected and categorized into responder and non-responder groups based on clinical response to mepolizumab. Responders were defined as patients who achieved a ≥ 50% reduction in oral corticosteroid use and/or a ≥ 50% reduction in exacerbation frequency after one year of treatment. Prior to data analysis, low-expression genes were filtered out. Genes with count values > 10 in at least three samples and an average count > 5 were retained for further analysis.

### Methods

#### Screening of differentially expressed genes

Differential expression analysis was conducted on the filtered dataset using the ‘DESeq2’ package (version 1.44.0)^10^ in R software (version 4.4.2). The parameter fitType = “mean” was applied, which fits the mean expression of each gene across groups to identify differentially expressed genes (DEGs) between the responder and non-responder groups. DEGs were defined as those with | log₂ fold change (log₂FC)| > 1 and raw P-value < 0.05. Genes with log₂FC > 1 and raw P-value < 0.05 were considered upregulated in the responder group, while those with log₂FC < − 1 and raw P-value < 0.05 were considered downregulated. The results of the differential expression analysis were visualized using the ‘ggplot2’ package (version 3.5.1)^11^.

#### Gene set enrichment analysis

Gene Set Enrichment Analysis (GSEA) is a statistical approach used to determine whether predefined gene sets are significantly enriched in gene expression data, thereby facilitating the identification of key pathways or biological processes associated with specific phenotypes or conditions. In this study, the Gene Ontology (GO) and Kyoto Encyclopedia of Genes and Genomes (KEGG) databases^12^ were selected as the sources of predefined gene sets. Pathway information was obtained from the KEGG database (Kanehisa Laboratories, www.kegg.jp/kegg/)。GSEA was performed in R using the ‘org.Hs.eg.db’ package (version 3.20.0)^13^, the ‘biomaRt’ package (version 2.62.0)^14^, the ‘clusterProfiler’ package (version 4.14.4)^15^,and the ‘fgsea’ package (version 1.32.2)^16^ to evaluate gene set enrichment between the two sample groups. The results were visualized using the ‘enrichplot’ package (version 1.26.5)^17^. Pathways with a false discovery rate (FDR q-value) < 0.05 and nominal P-value < 0.05 were considered statistically significant.

#### Construction of the protein–protein interaction (PPI) network and screening of core genes

The PPI network was constructed using the STRING database (Search Tool for the Retrieval of Interacting Genes/Proteins) with a minimum required interaction score of 0.4. Disconnected nodes were excluded from the network. The network was visualized using Cytoscape software (version 3.10.3). To further identify hub genes, four algorithms from the cytoHubba plugin—Degree, Maximum Clique Centrality (MCC), Maximum Neighborhood Component (MNC), and Edge Percolated Component (EPC)—were applied to rank genes in the PPI network. The top 10 genes identified by each algorithm were intersected to determine the core genes.

#### Validation of hub genes and evaluation of their diagnostic value

The R package ‘ggplot2‘(version 3.5.1) was used to generate scatter plots and box plots to compare the expression levels of hub genes between the responder and non-responder groups. A P-value less than 0.05 was considered statistically significant. Genes showing significant differential expression were further analyzed using the R package ‘pROC‘(version 1.18.5)^18^ to construct receiver operating characteristic (ROC) curves. The area under the curve (AUC) and 95% confidence intervals were calculated to evaluate the diagnostic performance of the hub genes.

## Results

### Data processing

After obtaining the expression matrix of the GSE274410 dataset, low-expression genes were filtered out based on the criteria that count values must exceed 10 in at least three samples and have an average count greater than 5. Of the 57,906 genes in the dataset, 19,908 genes were retained after filtering. Baseline clinical characteristics of the cohort are summarized in Supplementary Table S2, showing no significant differences between responders and nonresponders in terms of sex distribution, age, body mass index (BMI), comorbidities, or peripheral blood eosinophil counts. Notably, the cohort was characterized by obesity (mean BMI ~ 32), suggesting a predominance of the obese asthma phenotype, which should be considered when interpreting subsequent analyses.

### Differential expression analysis

**Fig. 1:**
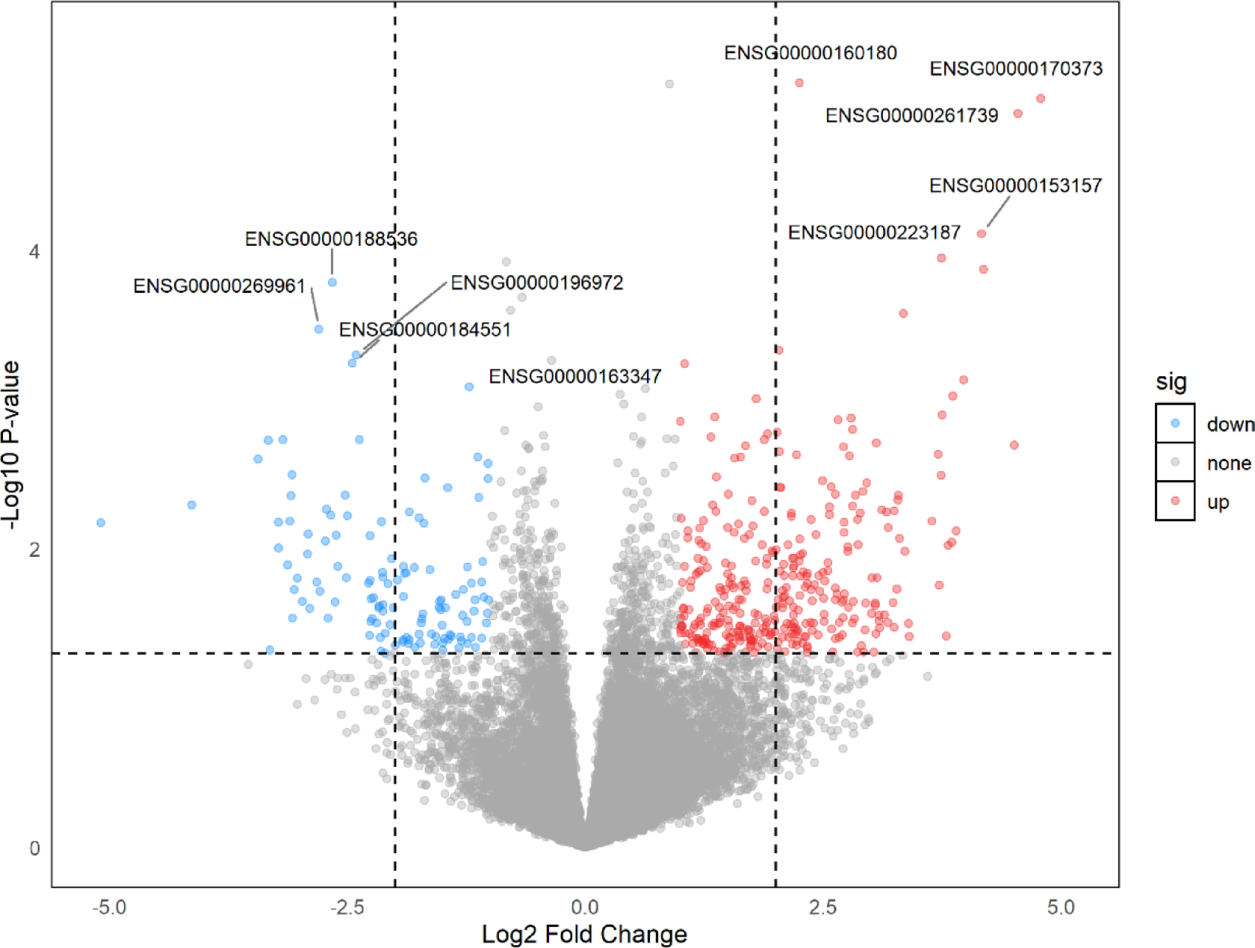
Volcano plot. Volcano plot showing differentially expressed genes (DEGs) between mepolizumab responders and non-responders. Each point represents a gene, with red dots indicating significantly upregulated genes, blue dots indicating significantly downregulated genes, and grey dots indicating non-significant genes. The x-axis represents log2 fold change (log2FC), and the y-axis represents –log10 adjusted p-value. Thresholds were set at raw p-value < 0.05 and |log2FC| > 1. Selected top DEGs are labeled with their Ensembl gene IDs.

Differential expression analysis was conducted on the 19,908 retained genes. According to the predefined criteria for DEGs (|log₂FC| > 1 and P-value < 0.05), a total of 467 DEGs were identified in the GSE274410 dataset, including 342 upregulated and 125 downregulated genes (Fig. 1).

### Gene set enrichment analysis

**Fig. 2:**
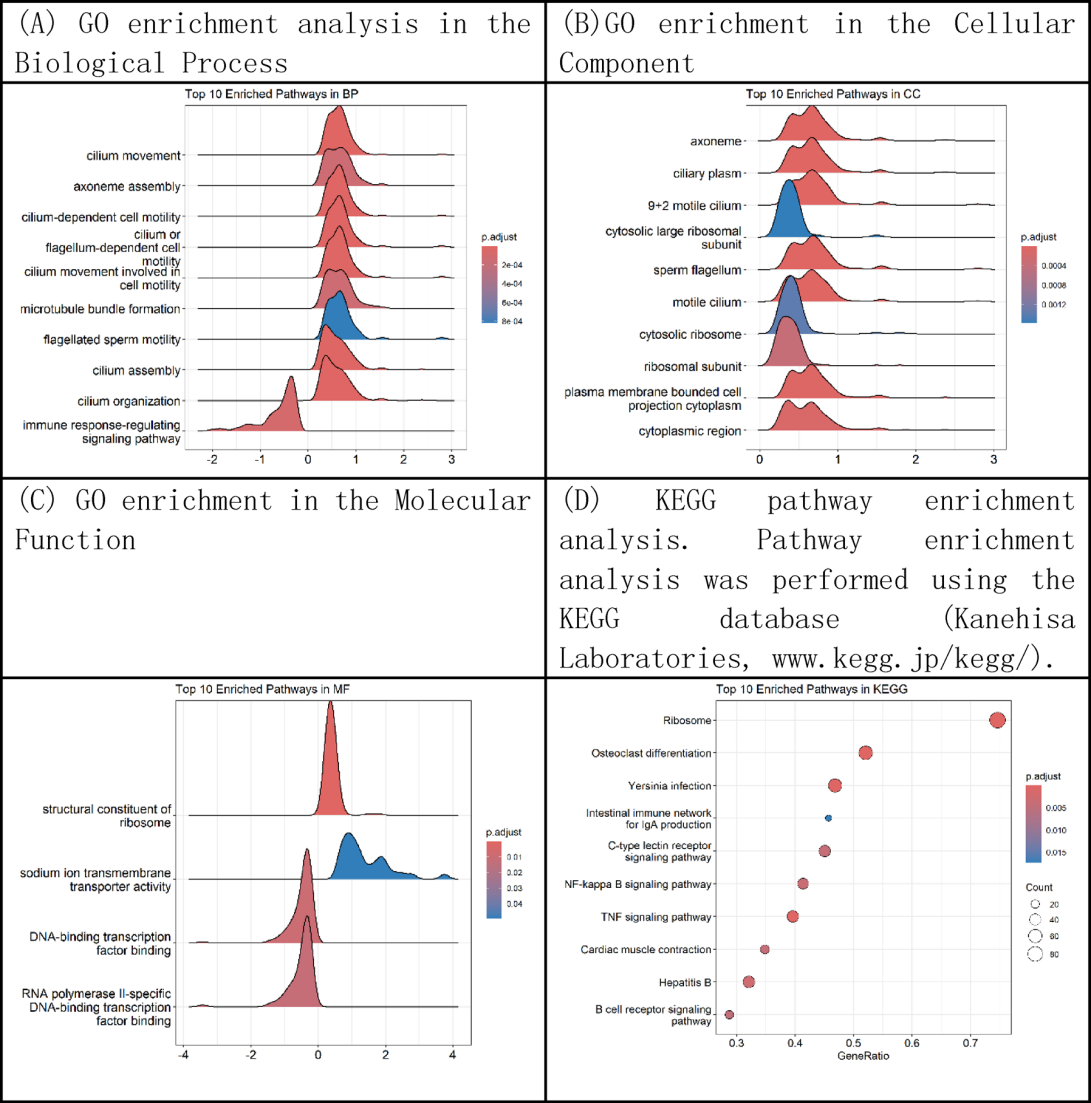
Gene enrichment analysis based on GO and KEGG using GSEA. **(A)** GO enrichment analysis in the Biological Process (BP) category, depicting pathways such as cilium movement, axoneme assembly, and flagellated sperm motility. **(B)** GO enrichment in the Cellular Component (CC) category, highlighting structures like axoneme, ciliary plasm, and cytosolic large ribosomal subunit.**(C) **GO enrichment in the Molecular Function (MF) category, including structural constituent of ribosome and transcription factor binding. **(D)** KEGG pathway enrichment analysis showing the top 10 significantly enriched pathways, including ribosome, IgA production, cardiac muscle contraction, and NF-kappa B signaling. Pathways with a false discovery rate (FDR q-value) < 0.05 and nominal P-value < 0.05 were considered statistically significant. Dot size reflects gene count, and color represents adjusted P-values.

GO enrichment analysis was performed to annotate and classify gene functions across three categories: Biological Process (BP), Cellular Component (CC), and Molecular Function (MF). GO and Kyoto Encyclopedia of Genes and Genomes (KEGG) enrichment analyses were conducted using the GSEA method based on the complete set of 19,908 genes. A total of 90 significantly enriched GO terms were identified, including 63 in the BP category, where the DEGs were primarily involved in ciliary-related processes such as *cilium movement*, *axoneme assembly*, *cilium- or flagellum-dependent cell motility*, and *cilium-dependent cell motility*. In the CC category, 23 significantly enriched terms were found, mainly associated with the *axoneme*, *ciliary plasm*, *9 + 2 motile cilium*, and *cytosolic large ribosomal subunit*. For the MF category, four significantly enriched terms were identified, including *structural constituent of ribosome*, *sodium ion transmembrane transporter activity*, *DNA-binding transcription factor binding*, and *RNA polymerase II-specific DNA-binding transcription factor binding*. KEGG pathway analysis revealed 20 significantly enriched pathways, including *Ribosome*, *Cardiac muscle contraction*, *Intestinal immune network for IgA production*, and *Allograft rejection* (Fig. 2).

### Construction of the PPI network and identification of core genes

**Fig. 3:**
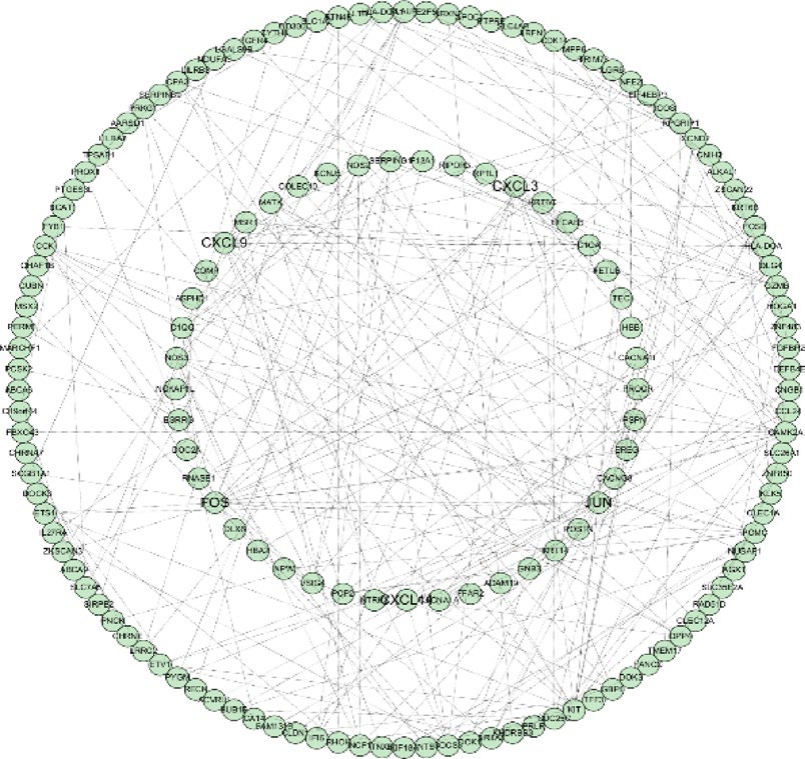
Protein–protein interaction (PPI) network. Protein–protein interaction (PPI) network of differentially expressed genes (DEGs). The network was constructed using the STRING database, and visualized in Cytoscape. Each node represents a protein encoded by a DEG, and edges represent predicted functional associations.

**Fig. 4:**
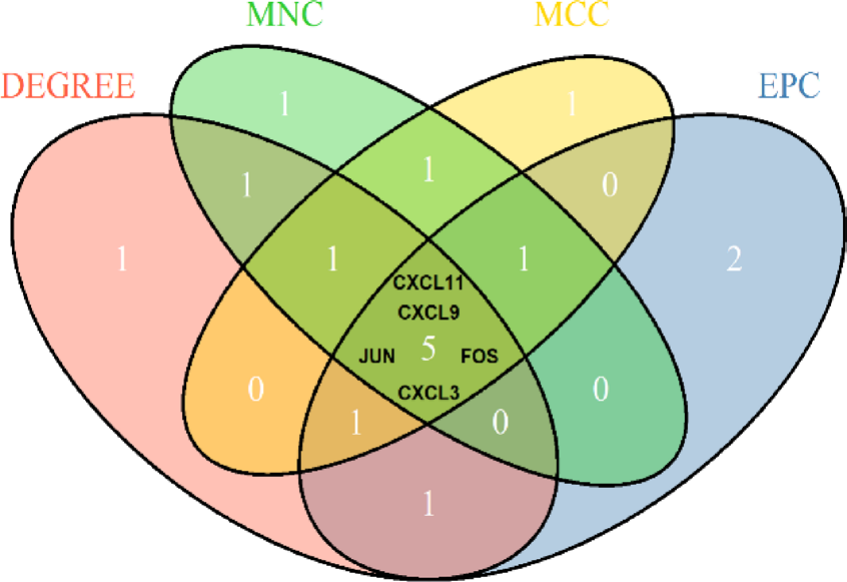
Venn diagram of overlapping hub genes from four cytoHubba algorithms. Venn diagram showing the overlap of hub genes identified by four topological algorithms (Degree, MNC, MCC, and EPC) in cytoHubba. Five genes (CXCL11, CXCL9, CXCL3, JUN, and FOS) were commonly identified across all four algorithms and selected as key hub genes.

To further elucidate protein-level interactions among the DEGs, a PPI network was constructed using the STRING online database. The resulting network was imported into Cytoscape software, yielding a structure comprising 155 nodes and 251 edges (Fig. 3). To identify hub genes within the network, four ranking algorithms from the cytoHubba plugin—Degree, Maximum Clique Centrality (MCC), Maximum Neighborhood Component (MNC), and Edge Percolated Component (EPC)—were applied. The top 10 genes identified by each algorithm are summarized in Supplementary Table 1. The intersection of the top 10 genes across all four algorithms was determined using the ‘VennDiagram’ package (version 1.7.3), resulting in five hub genes: *JUN*, *FOS*, *CXCL9*, *CXCL11*, and *CXCL3*(Fig. 4).

### Validation of core genes and evaluation of their diagnostic value

**Fig. 5:**
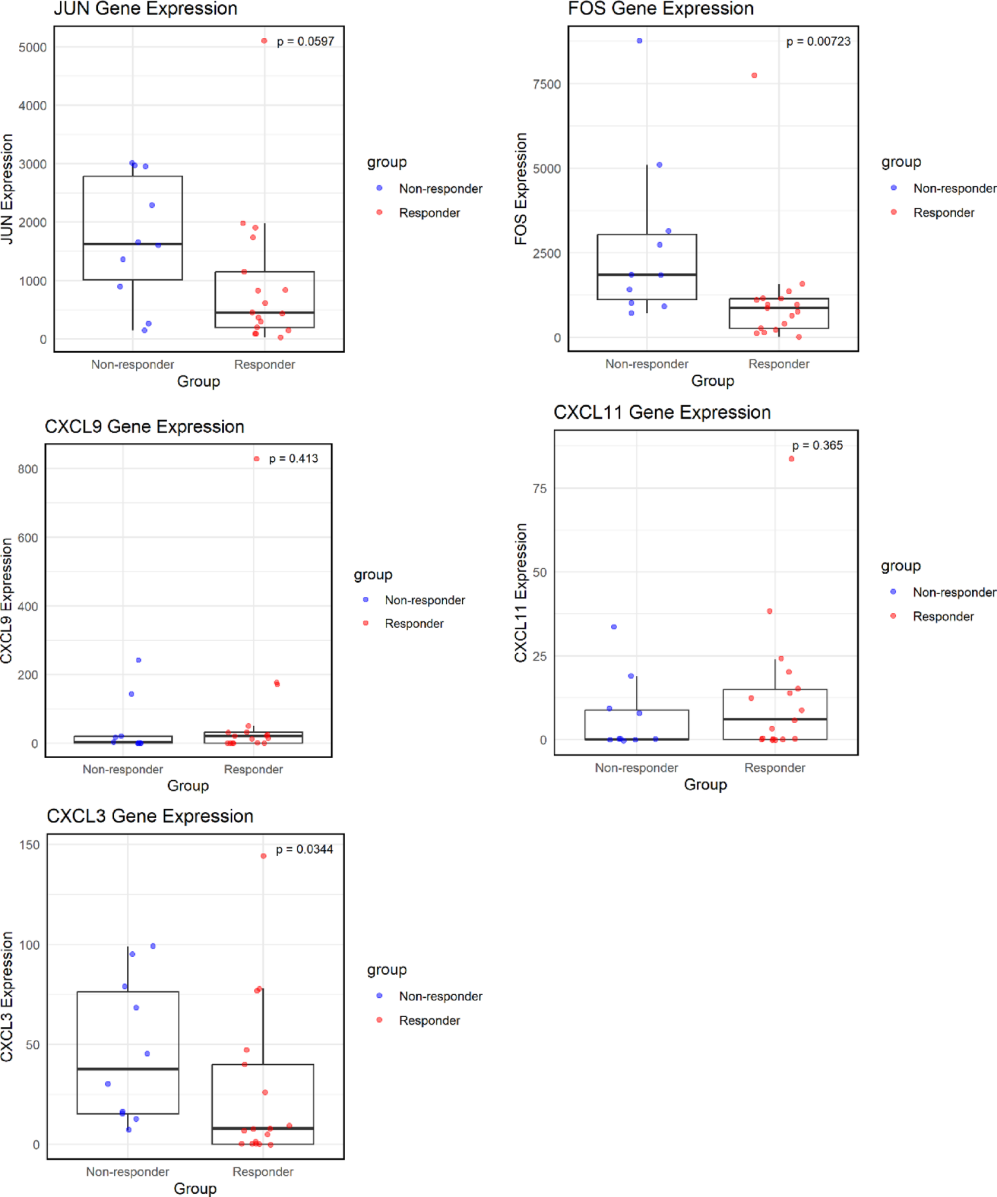
Comparison of core gene expression levels between groups. Comparison of expression levels of five hub genes between responder and non-responder groups. Boxplots represent the expression distributions of JUN, FOS, CXCL9, CXCL11, and CXCL3. Red indicates the responder group, and blue indicates the non-responder group. P-values were calculated to assess differential expression, A P-value < 0.05 was considered statistically significant.

**Fig. 6:**
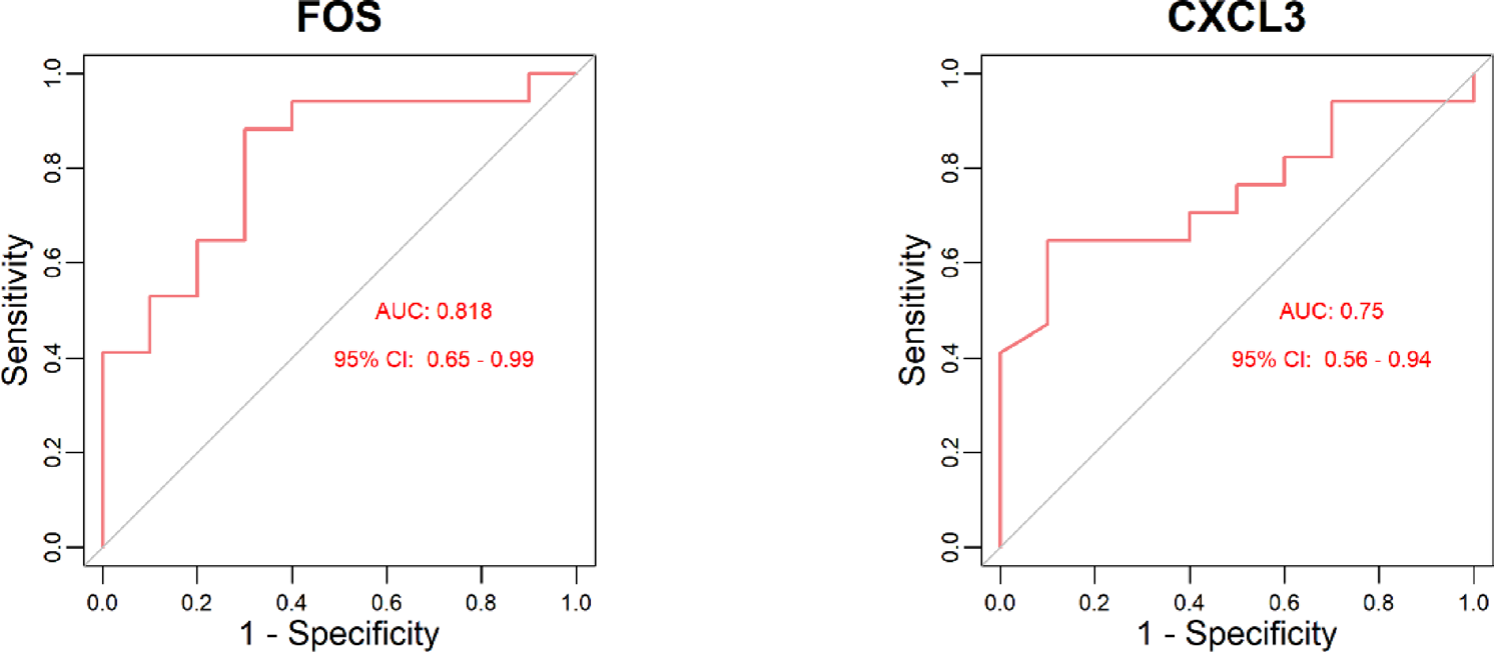
ROC curve of FOS and CXCL3. **(a)** FOS shows an AUC of 0.818 with 95% CI: 0.65–0.99. **(b)** CXCL3 shows an AUC of 0.75 with 95% CI: 0.56–0.94. Higher AUC values indicate better discriminatory ability between responder (*n* = 17) and non-responder (*n* = 10) groups.

To compare the expression levels of the hub genes (*JUN*, *FOS*, *CXCL9*, *CXCL11*, and *CXCL3*) between the responder and non-responder groups, scatter plots and box plots were generated using the ‘ggplot2’ package (version 3.5.1) in R, with P-values annotated on the plots (Fig. 5). The results showed that only *FOS* and *CXCL3* exhibited statistically significant differential expression between the two groups. To further assess their predictive value, ROC curves were constructed for *FOS* and *CXCL3* (Fig. 6). Both genes demonstrated area under the curve (AUC) values greater than 0.7, indicating good predictive performance.

## Discussion

In this study, we analyzed transcriptomic data from patients with severe eosinophilic asthma (GSE274410) to identify biomarkers associated with differential responses to mepolizumab therapy. A total of 467 differentially expressed genes (DEGs) were identified between responders and non-responders, and subsequent hub gene analysis highlighted FOS and CXCL3 as key candidates. These findings suggest potential molecular mechanisms underlying variability in treatment efficacy and provide novel insights into patient stratification for anti–IL-5 therapy.

In our analysis of transcriptomic data from patients with severe eosinophilic asthma, FOS and CXCL3 emerged as key candidate genes associated with differential responses to mepolizumab. Both genes were expressed at lower levels in responders, suggesting that they may serve as negative predictive biomarkers for treatment efficacy. The *FOS* gene encodes the c-Fos protein, a component of the Activator Protein-1 (AP-1) transcription factor complex that regulates inflammatory signaling and has been implicated in airway eosinophilic inflammation^19,20^. CXCL3 is a chemokine involved in neutrophil recruitment, airway inflammation, and remodeling^21,22^.Patients with low FOS and CXCL3 expression appeared more responsive to mepolizumab, which may indicate that higher expression reflects a neutrophil-skewed inflammatory profile less responsive to anti–IL-5, whereas lower expression is more consistent with a Th2-high, eosinophil-driven phenotype. This interpretation is speculative and requires further validation. Importantly, while these mechanisms have been previously described in asthma pathogenesis, our study is the first to highlight their predictive relevance for mepolizumab responsiveness. This finding suggests that transcriptomic biomarkers such as FOS and CXCL3 may provide complementary molecular insights beyond traditional clinical predictors, such as blood eosinophil counts^23^ and cytokine ratios^24^, thereby refining the framework for patient stratification in severe eosinophilic asthma.

GSEA enrichment analysis revealed that, at the GO Biological Process level, DEGs were significantly enriched in pathways such as *cilium movement*, *axoneme assembly*, *cilium- or flagellum-dependent cell motility*, and *cilium-dependent cell motility*. These pathways are primarily related to ciliary function, highlighting the pivotal role of ciliary motion in the pathogenesis of pulmonary diseases. It is noteworthy that a considerable proportion of these genes were associated with ciliated epithelial function. This likely reflects the sampling method, as nasal swabs predominantly capture superficial epithelial cells enriched in ciliated populations. Therefore, the transcriptional signatures observed here may represent epithelial surface biology, particularly ciliary activity, rather than deeper airway compartments. Previous studies have confirmed that proper ciliary function is essential for maintaining effective airway clearance^25^. In 2010, Biju Thomas et al. reported that bronchial epithelial cells from patients with severe asthma exhibited functional abnormalities, including reduced ciliary beat frequency and disorganized rhythmic motion. They further proposed that defects in ciliary structure and function may not merely be secondary to inflammation or infection, but rather represent an intrinsic pathological feature of asthma itself, particularly in severe forms of the disease^26^. These findings suggest that ciliary dysfunction is not merely a consequence of asthma, but also a potential contributor to airway mucus retention, chronic inflammation, and increased susceptibility to respiratory infections. Furthermore, studies have demonstrated a bidirectional interaction between mucus and eosinophils. Eosinophils can promote both the production and pathogenicity of mucus, while the presence of mucus can, in turn, exacerbate eosinophilic inflammation—particularly within the asthmatic airway^27^. Therefore, impaired mucociliary clearance resulting from structural ciliary abnormalities may exacerbate disease severity in patients with eosinophilic asthma and potentially influence their response to mepolizumab therapy. However, the underlying mechanisms of this association remain to be fully elucidated.

Interestingly, eosinophil-related and type 2 inflammation–associated gene signatures were not prominent in our analysis. This absence may be attributed to the fact that nasal epithelial transcriptomes primarily capture epithelial cell–intrinsic biology rather than immune cell–driven pathways that are more evident in bronchial tissue or peripheral blood. In addition, the relatively small cohort size may have limited statistical power to detect these signatures. It is also possible that responder–non-responder differences are mainly reflected in epithelial rather than systemic immune transcriptional programs. These considerations suggest that the biomarkers identified here may complement, rather than replace, classical type 2–related indicators such as peripheral blood eosinophil counts.

In addition, GSEA results revealed that DEGs were significantly enriched in the KEGG pathway *Intestinal immune network for IgA production*, suggesting that mucosa-associated immune responses may contribute to modulating the therapeutic efficacy of mepolizumab. In 1993, Alistair J. Ramsay et al. constructed a recombinant vaccinia virus vector expressing the murine *IL-5* gene and administered it intranasally to mice. The results showed a significant enhancement of pulmonary immunoglobulin A (IgA) responses, indicating that IL-5 plays an important role in mucosal IgA immunity^28^. IL-5 promotes mucosal IgA production by binding to the IL-5 receptor (IL-5R) α-chain. Studies have shown that in mice lacking IL-5Rα, the number of IgA-producing cells in mucosal effector sites—such as the intestinal lamina propria and nasal mucosa—is significantly reduced^29^. Taken together, these findings suggest that IL-5 plays a critical role in regulating mucosal IgA responses. Given that mepolizumab is an anti–IL-5 monoclonal antibody, it is plausible that its therapeutic efficacy may, at least in part, be mediated through modulation of IgA-associated mucosal immunity.

Several limitations of this study should be acknowledged. First, the transcriptomic data were derived from nasal epithelial cells, which, while practical and minimally invasive, may not fully represent lower airway biology. Second, the publicly available dataset (GSE274410) showed inconsistencies in participant numbers (27 vs. 29 based on smoking status) and lacked detailed clinical information, including medication history, intranasal corticosteroid use, and extended follow-up beyond three months, which is shorter than the recommended 6–12 month period for evaluating mepolizumab response^30^. These limitations, together with the predominance of an obese asthma phenotype, raise concerns about generalizability and may reduce the applicability of our findings to the broader severe eosinophilic asthma population. Third, the relatively small sample size limits statistical power, and potential confounders such as environmental exposures, seasonal variation, and concurrent medications could not be controlled for. Finally, it should be acknowledged that several of the biological mechanisms identified here—including CXCL3 and related chemokines, ciliary dysfunction, IgA-mediated mucosal immunity, and FOS gene expression—have been previously described in asthma research. The novelty of this work lies in applying these established mechanisms to the prediction of mepolizumab responsiveness. However, as the analysis was based on a single publicly available dataset (GSE274410) with a modest sample size, our conclusions remain exploratory and underscore the need for validation in independent datasets and larger, multi-center cohorts with richer clinical characterization.

In summary, low baseline expression of *FOS* and *CXCL3* in nasal epithelial surface cells may be associated with early responsiveness to mepolizumab treatment in patients with severe eosinophilic asthma, highlighting their potential as predictive biomarkers of therapeutic response. Early identification of likely responders prior to treatment initiation could facilitate the optimization of personalized therapeutic strategies, minimize unnecessary drug exposure, and reduce associated healthcare costs.

Unlike previous studies that primarily focused on clinical parameters such as blood eosinophil counts^23^ or cytokine ratios^24^, our study highlights transcriptomic biomarkers (FOS and CXCL3) that may provide complementary molecular insights into mepolizumab responsiveness. This distinction underscores the novelty of our findings and their potential value in refining patient stratification.

Future research should advance in the following directions. First, the diagnostic and predictive value of *FOS* and *CXCL3* should be validated in larger, multi-center clinical cohorts to assess their applicability and robustness across diverse populations. Second, the integration of single-cell sequencing, multi-omics analyses, and other high-resolution technologies may help uncover additional, more specific biomarkers and clarify their roles in immune regulation. Third, mechanistic studies using in vitro and in vivo models are needed to elucidate the regulatory pathways through which these genes modulate the response to anti–IL-5 therapy. Together, these future efforts will provide a theoretical foundation and potential molecular targets to support the development of more precise and individualized treatment strategies for asthma.

Collectively, our findings highlight the exploratory yet potentially breakthrough value of transcriptomic biomarkers such as FOS and CXCL3 in predicting mepolizumab responsiveness, underscoring their promise to complement established clinical markers and support precision medicine strategies in severe eosinophilic asthma.

## Supplementary Information

Below is the link to the electronic supplementary material.

Supplementary Material 1

Supplementary Material 2

Supplementary Material 3

## Data Availability

The R code used for analysis is available in the Supplementary Material 1. The datasets analyzed in this study are publicly available in the Gene Expression Omnibus (GEO) database under accession number GSE274410 (https://www.ncbi.nlm.nih.gov/geo/query/acc.cgi?acc=GSE274410).
