# Supplementary material for: Identification of key genes predicting the efficacy of mepolizumab in the treatment of severe eosinophilic asthma
Source: Sci Rep. 2025 Nov 12;15:39724. doi: 10.1038/s41598-025-23443-8 (PMC12612187; doi:10.1038/s41598-025-23443-8)
Supplement: Supplementary file 2 — Supplementary Material 2 [file 41598_2025_23443_MOESM2_ESM.docx]

**Supplementary Table S2. Patient Clinical and Demographic Features at Baseline and 12 Months in the GSE274410 dataset.**

| Baseline Nonresponder (*n* = 10) Responder (*n* = 17) *P* Value |
| --- |
| Sex  female, *n* (%) 8 (80) 9 (52.9) 0.16* |
| Age, yr 58.55 (44.58–68.96) 57.38 (38.24–64.58) 0.55^†^ |
| Body mass index 32.59 (9.33) 32.83 (6.17) 0.94^‡^ |
| Comorbidity,[^§^](javascript:popRef('TF6')) yes, *n* (%) 9 (90) 15 (88.2) 0.89* |
| Atopic rhinitis, *n* (%) 1 (10) 1 (5.9) 0.693* |
| Smoking, *n* (%) 0.35* Never 6 (60) 6 (35.3)  Ex 3(30) 10 (58.8)  Current 1(10) 1 (5.9) |
| Asthma control 3.42 (1.62) 3.27 (1.42) 0.81^‡^  questionnaire, average  score of 6 |
| Exacerbations per year 5.78 (3.23) 5.82 (2.40) 0.97^‡^ before recruitment |
| OCS at baseline, 16.80 (17.73) 8.82 (8.15) 0.121^‡^ prednisolone dose, mg |
| FEV_1_% predicted 58.65 (21.53) 69.79 (51.52) 0.24^‡^ |
| FEV_1_/FVC 65.83 (12.71) 68.64 (12.79) 0.61^‡^ |
| Eosinophils, highest in 0.5 (0.37–0.72) 0.50 (0.40–0.70) 0.51^†^  year before  recruitment, ×10^9^/L |
| White blood cells, ×10^9^/L 8.53 (6.85–9.29) 7.70 (6.36–10.78) 0.74^†^ |
| 12 months |
| Exacerbations per year, 5.14 (2.91) 1.64 (1.41) 0.02^‡^ 12 mo after recruitment |
| OCS at 12 mo, 20.00 (18.71) 4 (3.27) 0.003^‡^ prednisolone dose, mg |

*Notes:*

*Definition of abbreviation*: OCS = oral corticosteroids.

Data are presented as mean (SD) or median (interquartile range) unless otherwise specified.

* Chi-square test.

^†^ Mann-Whitney *U* test.

^‡^ Independent *t* test.

^§^ Comorbidities defined as anxiety/depression, cancer, deep vein thrombosis, diabetes, eczema, hay fever, hypertension, ischemic heart disease, pulmonary embolism, reflux, or renal failure.

Baseline characteristics and follow-up data were obtained from the GSE274410 dataset (original publication). The “All patients” column was omitted due to inconsistencies in the smoking variable when compared with individual-level data. Only limited 12-month follow-up information (exacerbations and oral corticosteroid use) was available in the GSE274410 dataset; other clinical characteristics were not collected beyond 3 months.
