# Supplementary material for: Identification of key genes predicting the efficacy of mepolizumab in the treatment of severe eosinophilic asthma
Source: Sci Rep. 2025 Nov 12;15:39724. doi: 10.1038/s41598-025-23443-8 (PMC12612187; doi:10.1038/s41598-025-23443-8)
Supplement: Supplementary file 3 — Supplementary Material 3 [file 41598_2025_23443_MOESM3_ESM.docx]

| Supplementary Table 1 Top 10 hub genes identified using four cytoHubba algorithms: Degree, MCC, MNC, and EPC. | |
| --- | --- |
| 算法 | 前10基因 |
| Degree | JUN KIT FOS CXCL9 CAMK2A CXCL11 POMC CXCL3 C1QA GZMB |
| MCC | JUN FOS CXCL9 C1QA CXCL11 C1QC POMC CXCL3 VSIG4 SOCS3 |
| MNC | JUN FOS CXCL9 C1QA CXCL11 CAMK2A DLG4 CXCL3 C1QC SOCS3 |
| EPC | JUN FOS CXCL9 SOCS3 KIT CXCL3 CXCL11 CCK NOS2 POM |

*Notes:*

Top 10 hub genes identified using four cytoHubba algorithms: Degree, MCC, MNC, and EPC. The table shows the top 10 ranked genes derived from each algorithm. CXCL11, CXCL9, CXCL3, JUN, and FOS were consistently identified across all methods, indicating their central role in the gene interaction network.
